# Scuba divers’ techniques to prevent fogging of eyewear in emergency healthcare settings

**DOI:** 10.3205/dgkh000347

**Published:** 2020-06-15

**Authors:** Abhirup Sarkar

**Affiliations:** 1 All India Institute of Medical Sciences, New Delhi, India

**Keywords:** PPE, fogging, goggles, Scuba

## Letter to the editor

Dear Editor,

The coronavirus disease 2019 (COVID-19) pandemic has caused an immense surge in the use of personal protective equipment (PPE) in healthcare settings. The use of goggles is an integral part of the PPE used by healthcare professionals involved in direct patient care [1], [2]. Fogging of goggles causing decreased visibility is a known issue faced by healthcare professionals everywhere [3]. This can often be catastrophic in critical stages of patient management.

Commercial anti-fogging agents commonly used by scuba divers can be used when donning goggles and have been found to be effective. Procuring sufficient amounts of commercially available anti-fogging agents can be difficult in the current situation. Some tricks used by professional scuba divers can be applied in emergency settings. Many scuba divers spit on the inner surface of their eyewear and wipe the sputum to prevent fogging [4]. We have used a similar technique in healthcare setting in emergency situations and found it to be effective. A better alternative, also used by scuba divers, is to use diluted shampoo or liquid soap to wipe the inner surface of the goggles before donning [4]. We found a 10% solution of shampoo or liquid soap solution was best at preventing fogging. A good practice is to put few drops of the solution on the inner surface of the glasses and rinse it with water. In emergency situations with no available soap solution, using the spit-and-wipe technique can be used to prevent fogging.

## Notes

### Competing interests

The author declares that he has no competing interests.
